# Longitudinal Psychological Distress After Malignant Brain Tumor Diagnosis: A Multilevel Analysis of Patients and Their Caregivers

**DOI:** 10.1002/pon.70064

**Published:** 2025-01-10

**Authors:** André Karger, Anna‐Maria Kisić, Caterina Quente, Maike K. Klett, Ralf Schäfer, Michael Sabel, Marion Rapp

**Affiliations:** ^1^ Clinical Institute of Psychosomatic Medicine and Psychotherapy Medical Faculty and University Hospital Düsseldorf Heinrich‐Heine‐University Düsseldorf Düsseldorf Germany; ^2^ Center for Integrated Oncology Aachen Bonn Cologne Düsseldorf (CIO ABCD) Düsseldorf Germany; ^3^ Department of Neurosurgery Medical Faculty and University Hospital Düsseldorf Heinrich‐Heine‐University Düsseldorf Düsseldorf Germany

**Keywords:** anxiety, brain tumor, cancer, caregiver, depression, distress, oncology, psycho‐oncology, psychological support

## Abstract

**Objective:**

Malignant brain tumors are associated with debilitating symptoms and a poor prognosis, resulting in high psychological distress for patients and caregivers. There is a lack of longitudinal studies investigating psychological distress in this group. This study evaluated fear of progression (FoP), anxiety and depression in patients and their caregivers in the 6 months following malignant brain tumor diagnosis.

**Methods:**

This prospective, observational study assessed FoP (FoP‐Q‐SF[P]), anxiety and depression (HADS) at diagnosis (T0) and after three (T1) and 6 months (T2) in patients with malignant brain tumors (primary, secondary) and their caregivers. Multilevel analyses were used to examine changes over time and differences between patients and caregivers, while accounting for the interdependence in their distress values.

**Results:**

Seventy‐one patients and 68 caregivers were included in the analysis. Throughout the study period, over 50% reported clinically relevant FoP, almost 50% reported clinically relevant anxiety, and over 30% reported relevant depression. Over all time points, caregivers reported significantly higher anxiety and depression than patients. Anxiety decreased between T0 and T2 in both groups. Exploratory analyses showed that female sex was associated with higher anxiety, and older age with higher depression. No significant predictors were identified for FoP.

**Conclusion:**

A substantial number of patients and caregivers experience clinically relevant psychological distress in the 6 months following a malignant brain tumor diagnosis. Caregivers are particularly distressed, reporting higher anxiety and depression. Integrating psycho‐oncological assessments and interventions for both patients and caregivers into clinical care is critical to address the psychological distress associated with malignant brain tumors.

## 1 Background

Malignant brain tumors (primary and secondary) are associated with particularly high psychological distress and burden for patients and their caregivers [1, 2, 3, 4], as therapy is mostly palliative and tumor progression is often inevitable [5, 6]. Prognosis in malignant brain tumors is generally poor, with an estimated 5‐year survival rate of 36% for primary brain tumors and a 2‐year survival rate of around 8.1% for secondary brain tumors [7, 8]. Typical patient symptoms include neurocognitive impairment, personality changes and seizures [9, 10], affecting patients and caregivers simultaneously [1, 11]. Patients and caregivers hope to prolong survival through combined therapy methods, while facing the fear of progression (FoP) and tumor recurrence [2, 12, 13].

While psychological distress in patients with malignant brain tumors and their caregivers is well documented [1, 2, 3, 4, 14], less is known about the progression of the distress over time within patients and their caregivers. Furthermore, most studies to date have only focused on primary brain tumors. For other cancer entities, research has shown that the psychosocial distress changes over the course of the disease both for patients and caregivers [15]. For patients with primary brain tumors post‐radiotherapy, Tibbs, Huynh‐Le [16] reported that anxiety decreased over the 12‐month study period, while depression remained unchanged. In glioma patients, high emotional distress was reported 8 weeks after neurosurgery and persisted at follow‐ups at three and 6 months [17]. Studies on caregivers of patients with brain tumors have mainly focused on associations between the caregivers' distress and patient characteristics. Finocchiaro, Petruzzi [18] found caregivers' psychosocial well‐being to depend on the disease duration and functional status of the patients. Another study on primary brain tumors found that caregivers' burden at diagnosis was associated with lower social support at 4 months, which in turn was associated with higher anxiety and depression in caregivers at 8 months [19]. These findings highlight the importance of providing timely support for distressed individuals.

The caregiver represents an important psychosocial resource for the patient. Patient and caregiver stress and coping have been described as interpersonal processes that affect both parties simultaneously. Therefore, cancer is often referred to as a “we‐disease” [20]. In lung cancer patients, caregiver health problems predicted subsequent distress in both patients and caregivers [21]. Furthermore, patients with primary brain tumors themselves reported their caregivers to be faced with diverse responsibilities [22]. Of note Goebel, von Harscher [1] indicated that partners of patients with primary brain tumors might be more affected than patients, as they more frequently exhibited psychiatric disorders within the first 3 months of diagnosis. These findings not only highlight the significant psychological distress of patients and caregivers, but also emphasize the potential negative impact of caregiver distress on patients themselves. However, few studies evaluated patients and their respective caregivers in a single study. Furthermore, to our knowledge, no study evaluated the psychological distress in dyads of patients with brain tumors and caregivers in a longitudinal study. However, longitudinal observations are necessary to better understand the detailed course of the psychological distress both in patients and caregivers.

Therefore, this study aimed to evaluate FoP, anxiety and depression in patients with primary and secondary malignant brain tumors and their caregivers from immediately after diagnosis (and primary neurosurgery) to 6 months post‐diagnosis. Given the poor prognosis for the vast majority of patients, we hypothesized that (1) the psychological distress persists or increases in both patients and caregivers. Moreover, we postulated that (2) caregivers are at least as affected by psychological distress as the patients themselves in the total sample. Additionally, we assessed whether sex, age and progression status influenced the psychological distress of participants over time.

## 2 Methods

### 2.1 Study Design

In this prospective, observational, monocentric study, patients with malignant brain tumors and their primary caregivers were recruited from July, 2019 to August, 2020 at the Department of Neurosurgery, University Hospital Duesseldorf. The present study is reported in accordance with the Strengthening the Reporting of Observational Studies in Epidemiology (STROBE) Statement [23] and registered in the German Clinical Trials Register (DRKS00034637).

The study was approved by the ethics committee of the Medical Faculty at Heinrich‐Heine‐University Duesseldorf (ID: 2018‐338‐ProspDEuA). Participants gave written informed consent and withdrawal was possible at any time.

### 2.2 Setting

The Department of Neurosurgery provided the study team with a list of all patients who were scheduled for brain tumor surgery. All accessible patients and their caregivers were visited by the study team on the ward three to 7 days after the operation, informed about the study and checked for inclusion criteria. If consent was obtained, patients and their caregivers were asked to complete self‐assessment questionnaires for FoP, anxiety and depression. The time points for the assessments were three to 7 days (baseline; T0), 3 months (T1) and 6 months (T2) post‐diagnosis. The T1 and T2 time points were chosen based on the regular scheduled visits at the Department of Neurosurgery.

### 2.3 Participants and Procedures

Inclusion criteria were (1) first diagnosis and elective admission for primary neurosurgery of a malignant brain tumor (primary and secondary), (2) age ≥ 18 years, (3) legal capacity, (4) informed consent, (5) further adjuvant treatment at the Department of Neurosurgery, (6) caregiver consent to participate and (7) sufficient proficiency in German. Patients were excluded if they: (1) had a severe aphasic disorder, (2) were under terminal palliative care (prognosis < 3 months) and (3) had physical or cognitive impairments preventing them from completing the study. Caregivers were considered as “primary care providers,” that is, the one who primarily takes care of the patient during his/her disease [18]. Patients indicated their primary caregiver.

### 2.4 Variables

FoP in patients was assessed using the short version of the *Fear of Progression‐Questionnaire* (FoP‐Q‐SF [24]). Caregivers filled out the validated FoP questionnaire for partners of chronically ill patients (FoP‐Q‐SF‐P [25]). The questionnaires have good reliability with a Cronbach's *α* of 0.87 [24] and 0.88 [25]. They consist of 12 items referring to four subscales: affective reactions, partnership/family, occupation, and loss of autonomy. Participants rate each item on a five‐point Likert scale (from 1–*never* to 5–*always*). The total score represents the extent of FoP, with higher scores indicating greater FoP. Referring to Mehnert, Berg [26], we defined a score of ≥ 4 (*often* and *very often*) on at least 50% of the items (total score ≥ 30) as clinically relevant moderate FoP, and on at least 75% of the items (total score ≥ 39) as high FoP.

The German version of the *Hospital Anxiety and Depression Scale* (HADS [27]) was used for the evaluation of anxiety and depression. The 14‐item self‐report questionnaire consists of 7 items each for anxiety (HADS‐A) and depression (HADS‐D), with four‐point Likert scales. A Cronbach's *α* between 0.82 and 0.90 has been evaluated [28]. A cut‐off score between 13 and 18 is recommended for various cancer entities [29]. For the subscales, a cut‐off score of ≥ 8 was employed to define a clinically relevant screening result [30]. Anxiety and depression scores between 8 and 10 were interpreted as mild, between 11 and 15 as moderate and between 16 and 21 as severe [31].

*Sociodemographic* (age, sex, housing situation, German citizenship, relationship status, children, educational level, employment, relationship with patient/caregiver, psychotropic drug intake, psychotherapy) and *medical characteristics* (functional status, type of cancer diagnosis, treatment, disease progression) of patients were partly extracted from health records, while caregivers were requested to complete a sociodemographic questionnaire.

### 2.5 Statistical Analysis

Descriptive statistics for all sociodemographic and disease‐specific data for patients and their caregivers were presented using frequencies, means and standard deviations. To investigate potential bias due to a high drop‐out rate, a drop‐out analysis was performed. To test hypotheses 1 and 2, multilevel analyses were calculated to account for the interdependence of data between patients and caregivers. Our model therefore included the random intercept *dyads*. All available data were included in the analyses, even if participants did not complete the questionnaires at all time points or if caregiver questionnaires were missing for patients. Outliers with an absolute z‐score value greater than 3.29 were removed, as this cut‐off value is a conventional criterion for identifying significant outliers [32]. The model chosen for data analysis included the fixed factors group (patient, caregiver), time (T0, T1, T2) and the interaction between group and time. The regression equation was as follows:

Outcome=RandomIntercept+bxGroup+bxTime+bxGroup:Time+Residual

For the exploratory analyses, sex, age and progression status (stable vs. progressive) were included as predictors in the final models. Here, a reduced data set was used, as some sociodemographic information was missing for certain participants. This approach ensured that the results of the main research question were based on the maximum available sample size, while the influence of missing values was taken into account in the exploratory analyses. Statistical analyses were performed using R Statistical Software (Version 4.3.2). An alpha level of 0.05 was considered statistically significant.

## 3 Results

### 3.1 Participants

**FIGURE 1:**
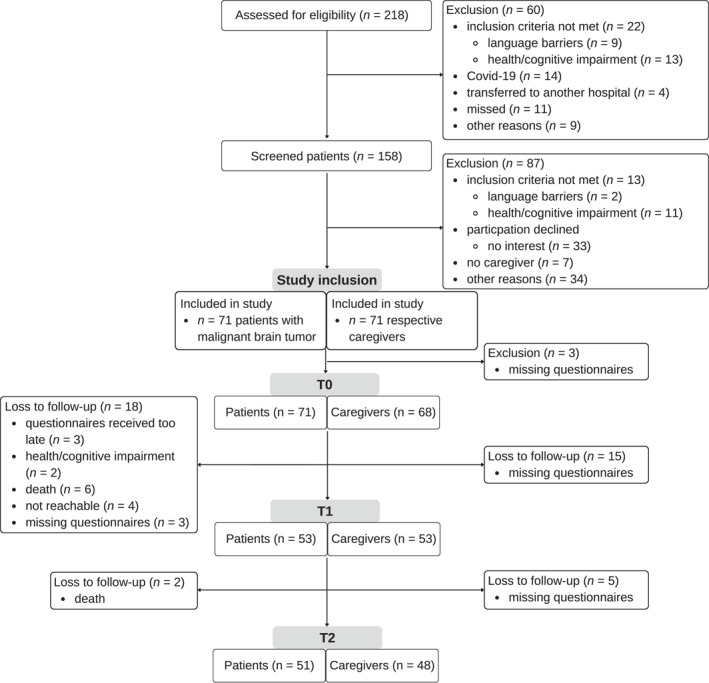
Flow diagram.

Two hundred and eighteen patients with malignant brain tumors were potentially eligible for study participation. In total, 71 patients and their caregivers were recruited. In the analysis, the data of 71 patients and 68 caregivers at baseline were included, with drop‐outs per time point depicted in Figure 1.

In most patients (*n* = 49, 69.0%), a glioma (diffuse glioma, WHO II, *n* = 11; anaplastic glioma/glioblastoma, WHO III/IV, *n* = 38) was diagnosed, while in 22 patients (31.0%), cerebral metastases were identified. Tumor location was mainly fronto‐temporal (frontal: *n* = 29, temporal: *n* = 17, parietal: *n* = 13, occipital: *n* = 4, cerebellar: *n* = 8, left‐sided: *n* = 35, right‐sided: *n* = 32). At each time point, 43 patients had a stable tumor type and 20 patients a progressive tumor type (*n* = 8 without tumor type information). All patients were undergoing systemic and/or local therapy during the study period. Caregivers were predominantly partners followed by family members. Detailed sociodemographic and clinical data are summarized in Table 1.

**TABLE 1:** Demographic and disease‐related data from patients and their caregivers at T0.

	Patients (*n* = 71)		Caregivers (*n* = 68)	
	*n* (missing)	%	*n* (missing)	%
Age	71 (0)	100 (0)	65 (3)	95.6 (4.4)
Mean; SD	56.3	15.1	53.9	15.5
Sex	71 (0)	100 (0)	65 (3)	95.6 (4.4)
Women	35	49.3	40	61.5
Men	36	50.7	25	38.5
Housing situation	70 (1)	98.6 (1.4)	66 (2)	97.1 (2.9)
Same household	50	71.4	46	69.7
Other household	9	12.9	15	22.7
Alone	11	15.7	5	7.6
German citizenship	70 (1)	98.6 (1.4)	65 (3)	95.6 (4.4)
Yes	58	82.9	51	78.5
Relationship status	71 (0)	100 (0)	68 (0)	100 (0)
Married, in partnership	57	80.3	61	89.7
Unmarried, widowed	14	19.7	7	10.3
Children	69 (2)	97.2 (2.8)	66 (2)	97.1 (2.9)
Yes	47	68.1	44	66.7
< 18 years	13	18.8	14	21.2
≥ 18 years	34	49.3	30	45.5
Educational level	70 (1)	98.6 (1.4)	65 (3)	95.6 (4.4)
< 12 years	34	47.9	29	42.6
≥ 12 years	36	50.7	36	53.0
Employment	68 (3)	95.8 (4.2)	63 (5)	92.6 (7.4)
Working	25	36.8	36	57.1
Retired	29	42.6	22	34.9
Not working	14	20.6	5	8.0
Relationship with patient/caregiver	71 (0)	100 (0)	68 (0)	100 (0)
Partner	48	67.6	46	67.6
Family member	19	26.8	18	26.5
Other (e.g. friend)	4	5.6	4	5.9
Psychotropic drug intake	70 (1)	98.6 (1.4)	59 (9)	86.8 (13.2)
Yes	10	14.3	5	8.5
No	59	84.3	54	91.5
Don't know	1	1.4	0	0
Psychotherapy (current/previous)	66 (5)	93.0 (7.0)	59 (9)	86.8 (13.2)
Yes	13	19.7	12	20.3
No	52	78.8	46	78.0
Don't know	1	1.5	1	1.7
Functional status	71 (0)	100 (0)		
ECOG 0–2	67	94.4		
ECOG 3–4	4	5.6		
Diagnosis	71 (0)	100 (0)		
Brain tumor	49	69.0		
Metastasis	22	31.0		

*Note:* Distinguishing at group level rather than dyadic level resulted in different sample sizes for patients and caregivers due to missing data.

Abbreviation: SD = standard deviation.

Drop‐out analyses did not show differences between the initial group and the study group in terms of sociodemographic data, FoP, anxiety and depression (all outcomes *t*(69) < 1.58, all outcomes *p* > 0.123).

### 3.2 Descriptive Statistics for Patients and Caregivers

**FIGURE 2:**
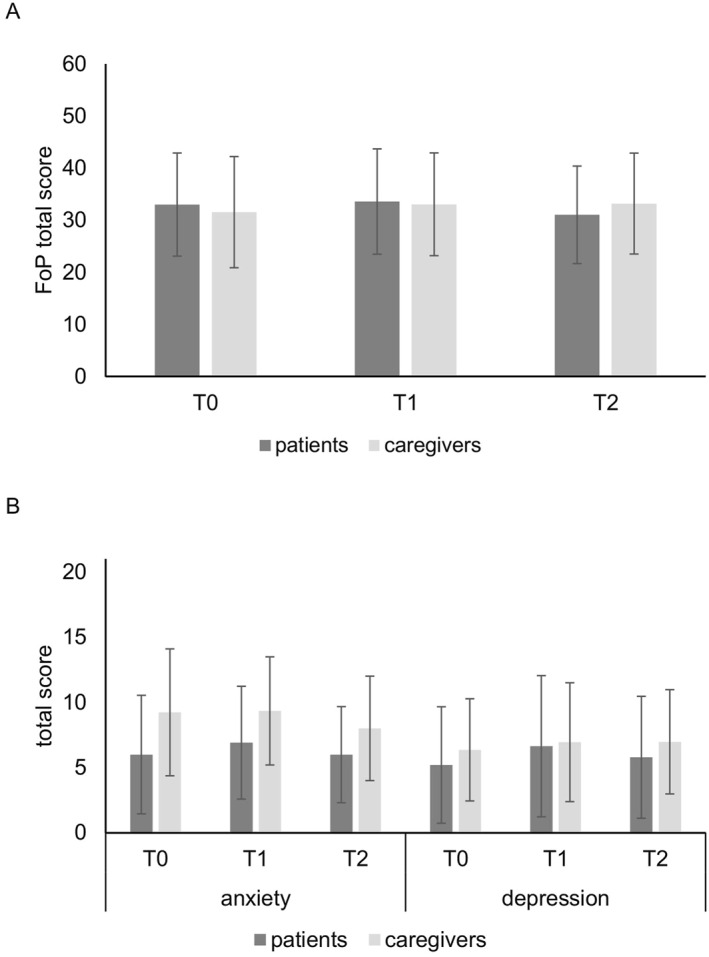
Means of fear of progression, anxiety and depression at all time points for patients and caregivers. T0: T0 = post‐diagnosis; T1 = after 3 months; T2 = after 6 months. Error bars represent standard deviations. The maximum FoP score to be achieved is 60. The maximum score for the subscales anxiety and depression subscales is 21. FoP = fear of progression.

Overall, over 50% of participants reported clinically relevant FoP (T0: 58.5%, T1: 65.4%, T2: 58.8%), nearly 50% reported clinically relevant anxiety (T0: 46.6%, T1: 50.0%, T2: 45.9%), and over 30% reported relevant depression (T0: 31.5%, T1: 42.6%, T2: 36.1%). More patients tended to have clinically relevant FoP than caregivers at T0 and T1, whereas the proportion of caregivers with clinically relevant FoP was greater than that of patients at T2. Looking at anxiety over all time points, a higher percentage of caregivers indicated clinically relevant scores compared with patients. Severe anxiety was reported by around 5%–10% of caregivers, while patients experienced mostly mild and moderate anxiety. Regarding depression, a higher proportion of caregivers indicated clinically relevant values compared with the proportion of patients, although the distribution of severity was relatively similar (see Table 2). See Figure 2 for a graphical representation of the descriptive statistics.

**TABLE 2:** Descriptive statistics of fear of progression, anxiety and depression.

	T0		T1		T2	
	Patients	Caregivers	Patients	Caregivers	Patients	Caregivers
FoP	*n* = 67	*n* = 63	*n* = 51	*n* = 53	*n* = 51	*n* = 46
Mean	33.01	31.56	33.59	33.06	31.04	33.20
SD	9.89	10.67	10.10	9.86	9.36	9.68
≥ 30	59.70%	57.14%	70.59%	60.38%	56.52%	60.78%
Moderate	28.36%	26.98%	33.34%	32.08%	30.43%	29.41%
High	31.34%	30.16%	37.25%	28.30%	26.09%	31.37%
HADS‐A	*n* = 70	*n* = 61	*n* = 52	*n* = 50	*n* = 51	*n* = 47
Mean	5.99	9.23	6.90	9.34	5.98	8.00
SD	4.55	4.87	4.33	4.15	3.69	4.01
≥ 8	31.43%	63.93%	36.54%	64.00%	35.29%	57.45%
Mild	12.86%	19.67%	11.54%	26.00%	23.53%	34.04%
Moderate	12.86%	34.43%	25.00%	28.00%	11.76%	19.15%
Severe	5.71%	9.83%	0.00%	10.00%	0.00%	4.26%
HADS‐D	*n* = 69^a^	*n* = 60	*n* = 52	*n* = 49	*n* = 51	*n* = 46
Mean	5.19	6.35	6.63	6.94	5.78	6.97
SD	4.47	3.92	5.42	4.56	4.68	4.00
≥ 8	25.71%	38.34%	40.38%	44.92%	39.21%	32.60%
Mild	7.14%	25.00%	15.38%	26.53%	25.49%	17.39%
Moderate	17.14%	11.67%	19.23%	12.24%	11.76%	13.04%
Severe	1.43%	1.67%	5.77%	6.12%	1.96%	2.17%

*Note:* FoP: Moderate 30–38, High ≥ 39, cut‐off for clinical significance ≥ 30; HADS‐A and HADS‐D: Mild 8–10, Moderate 11–15, Severe 16–21, cut‐off for clinical significance ≥ 8.

Abbreviations: FoP = fear of progression, HADS‐A = hospital anxiety and depression scale—anxiety, HADS‐D = hospital anxiety and depression scale—depression, SD = standard deviation.

^a^ 1 Outlier was removed (*z* > 3.29).

### 3.3 Multilevel Analyses Regarding Fear of Progression, Anxiety and Depression

Looking at FoP, none of the fixed effects emerged as significant predictors. Table 3 shows the multilevel modeling results for FoP.

**TABLE 3:** Fixed and random effect estimates of the multilevel models.

Outcome	Parameter	Estimates (SE)	CI	*t*	*p*‐value
FoP	*Fixed effects*				
	Intercept	31.35 (1.25)	[28.91; 33.80]	25.04	**< 0.001**
	Group	1.82 (1.49)	[−1.10; 4.73]	1.22	0.225
	Time T0 versus T1	1.49 (0.72)	[−1.62; 4.61]	0.94	0.351
	Time T0 versus T2	1.40 (0.73)	[−1.74; 4.55]	0.87	0.384
	Group:T1	−0.36 (2.25)	[−4.76; 4.03]	−0.16	0.872
	Group:T2	−2.86 (2.30)	[−7.34; 1.62]	−1.24	0.215
	*Random effects*	Variance (SD)			
	Intercept (between dyads)	28.85 (5.37)			
	Residual (within groups)	69.93 (8.36)			
	Marginal/Conditional *R* ^2^	0.01/0.30			
	Observations	331			
	Dyads	71			
HADS‐A	*Fixed effects*				
	Intercept	9.25 (0.54)	[8.19; 10.31]	16.90	**< 0.001**
	Group	−3.25 (0.59)	[−4.40; −2.11]	−5.37	**< 0.001**
	Time T0 versus T1	−0.11 (0.65)	[−1.37; 1.15]	−0.16	0.869
	Time T0 versus T2	−1.36 (0.66)	[−2.65; −0.08]	−2.07	**0.040**
	Group:T1	0.83 (0.87)	[−0.89; 2.55]	0.94	0.349
	Group:T2	1.29 (0.88)	[−0.45; 3.04]	1.45	0.149
	*Random effects*	Variance (SD)			
	Intercept (between dyads)	7.49 (2.74)			
	Residual (within groups)	10.82 (3.29)			
	Marginal/Conditional *R* ^2^	0.10/0.47			
	Observations	331			
	Dyads	71			
HADS‐D	*Fixed effects*				
	Intercept	6.50 (0.56)	[5.41; 7.60]	11.61	**< 0.001**
	Group	−1.41 (0.59)	[−2.57; −0.25]	−2.38	**0.018**
	Time T0 versus T1	0.31 (0.65)	[−0.97; 1.59]	0.47	0.636
	Time T0 versus T2	−0.36 (0.67)	[−1.66; 0.94]	−0.54	0.588
	Group:T1	1.15 (0.89)	[−0.59; −2.90]	1.29	0.197
	Group:T2	1.22 (0.90)	[−0.54; 2.98]	1.36	0.176
	*Random effects*	Variance (SD)			
	Intercept (between dyads)	8.40 (2.90)			
	Residual (within groups)	10.86 (3.30)			
	Marginal/Conditional *R* ^2^	0.02/0.45			
	Observations	327			
	Dyads	71			

Abbreviations: CI = confidence interval (0.95), FoP = fear of progression, HADS‐A = hospital anxiety and depression scale—anxiety, HADS‐D = hospital anxiety and depression scale—depression, SD = standard deviation, SE = standard error.

For anxiety, group (*b* = −3.25, *t*(255) = −5.54, *p* < 0.001) and time (*b* = −1.36, *t*(255) = −2.07, *p* = 0.040) were significant predictors in our model, while no interaction was observed. Caregivers indicated significantly higher values of anxiety than patients. For time, there was a significant effect on anxiety, with lower anxiety values at T2 than at T0. Table 3 gives a detailed overview over the multilevel modeling results for anxiety.

In terms of depression, the only significant predictor was group (*b* = −1.41, *t*(251) = −2.38, *p* = 0.013), while time and interaction showed no significant effect on depression (see Table 3). Caregivers showed significantly higher depression values than patients at all time points.

The exploratory multilevel analyses showed no influence of the sociodemographic predictors, age, sex and progression status for FoP. For anxiety, group and sex (*b* = 0.85, *t*(221) = 2.03, *p* = 0.044) were significant predictors. Caregivers indicated higher values of anxiety than patients and women showed significantly higher anxiety values than men. With regard to depression, group and age (*b* = 0.09, *t*(215) = 4.76, *p* < 0.001) emerged as significant predictors. Being a caregiver and older age were significantly associated with higher values of depression. The detailed values for the exploratory models are shown in Table 4.

**TABLE 4:** Fixed and random effect estimates of the exploratory models.

Outcome	Parameter	Estimates (SE)	CI	*t*	*p*‐value
FoP	*Fixed effects*				
	Intercept	33.42 (2.91)	[27.77; 39.06]	11.48	**< 0.001**
	Group	0.86 (1.67)	[−2.37; 4.09]	0.52	0.605
	Time T0 versus T1	1.32 (1.77)	[−2.11; 4.75]	0.75	0.455
	Time T0 versus T2	1.36 (1.82)	[−2.16; 4.89]	0.75	0.454
	Group:T1	−0.56 (2.47)	[−5.34; 4.22]	−0.23	0.821
	Group:T2	−3.64 (2.53)	[−8.56; 1.27]	−1.44	0.152
	Sex	−0.39 (1.14)	[−2.60; 1.83]	−0.34	0.735
	Age	−0.01 (0.05)	[−0.10; 0.08]	−0.19	0.850
	Progress	−0.72 (1.43)	[−3.53; 2.09]	−0.51	0.615
	*Random effects*	Variance (SD)			
	Intercept (between dyads)	24.79 (4.98)			
	Residual (within groups)	68.93 (8.30)			
	Marginal/Conditional *R* ^2^	0.01/0.27			
	Observations	275			
	Dyads	61			
HADS‐A	*Fixed effects*				
	Intercept	8.66 (1.22)	[6.30; 11.01]	7.12	**< 0.001**
	Group	−3.05 (0.61)	[−4.24; −1.86]	−4.98	**< 0.001**
	Time T0 versus T1	−0.47 (0.67)	[−1.77; 0.84]	−0.70	0.488
	Time T0 versus T2	−1.17 (0.69)	[−2.50; 0.16]	−1.70	0.090
	Group:T1	0.91 (0.92)	[−0.88; 2.69]	0.99	0.326
	Group:T2	0.98 (0.93)	[−0.83; 2.79]	1.05	0.294
	Sex	0.85 (0.42)	[0.04; 1.67]	2.03	**0.044**
	Age	0.01 (0.02)	[−0.03; 0.04]	0.26	0.796
	Progress	0.36 (0.70)	[−1.02; 1.73]	0.51	0.612
	*Random effects*	Variance (SD)			
	Intercept (between dyads)	6.86 (2.62)			
	Residual (within groups)	10.18 (3.19)			
	Marginal/Conditional *R* ^2^	0.11/0.47			
	Observations	289			
	Dyads	61			
HADS‐D	*Fixed effects*				
	Intercept	1.52 (1.19)	[−0.79; 3.82]	1.28	**< 0.001**
	Group	−1.59 (0.63)	[−2.82; −0.37]	−2.52	**0.013**
	Time T0 versus T1	0.25 (0.70)	[−1.10; 1.60]	0.36	0.723
	Time T0 versus T2	−0.23 (0.71)	[−1.60; 1.14]	−0.32	0.748
	Group:T1	0.97 (0.95)	[−0.87; 2.82]	1.02	0.307
	Group:T2	1.22 (0.96)	[−0.63; 3.08]	1.28	0.203
	Sex	−0.09 (0.43)	[−0.92; 0.74]	−0.22	0.829
	Age	0.09 (0.02)	[0.06; 0.13]	4.76	**< 0.001**
	Progress	0.69 (0.65)	[−0.59; 1.98]	1.06	0.294
	*Random effects*	Variance (SD)			
	Intercept (between dyads)	5.12 (2.26)			
	Residual (within groups)	10.55 (3.25)			
	Marginal/Conditional *R* ^2^	0.14/0.42			
	Observations	283			
	Dyads	61			

Abbreviations: CI = confidence interval (0.95), FoP = fear of progression, HADS‐A = hospital anxiety and depression scale—anxiety, HADS‐D = hospital anxiety and depression scale—depression, SD = standard deviation, SE = standard error.

## 4 Discussion

The aim of this study was to evaluate FoP, anxiety and depression in patients with primary and secondary malignant brain tumors and their caregivers at diagnosis to 6 months follow‐up. Our results suggest that a substantial group of both patients and caregivers have clinically relevant FoP, anxiety and depression. Interestingly, caregivers reported even greater anxiety and depression than patients at all time points. Previous literature on FoP, anxiety and depression in the subgroup of patients with primary and secondary malignant brain tumors is sparse. Gibson and Graber [33] estimated the prevalence of cancer‐related distress and psychiatric disorders in patients with primary and secondary brain tumors to range between 38% and 48%. In our study, we observed anxiety and depression rates of 31% and 26% in patients at baseline (percentages of patients over the cut‐off), however only cautious comparisons between psychiatric disorders and self‐reported symptoms are possible. Our baseline results for FoP in patients (mean 33) are comparable to those of Goebel and Mehdorn [12] (mean 32), who assessed FoP only in patients with primary brain tumors at an early treatment phase. For caregivers, our rates of clinically relevant anxiety (64%) and depression (38%; percentages of participants over cut‐off), align with those of Stieb, Fischbeck [14], who assessed caregivers of patients with primary brain tumors at varying time points during the patient's disease course (applying the same cut‐off: anxiety 71%, depression 40%). For caregivers of patients with secondary brain tumors, similar rates of anxiety (61%) have been reported, but depression values were reported to be higher than in our sample (52%; using the same assessment tools and thresholds [34]). This discrepancy may have resulted from our diverse population of primary and secondary brain tumor patients. To our knowledge, only Braun, Aslanzadeh [13] assessed FoP in caregivers, but used a different instrument. Moreover, no specific data currently exist on FoP among caregivers or patients specifically dealing with secondary brain tumors. Overall, the observed psychological distress in this study aligns with findings reported in the literature.

Consistent with our primary hypothesis, we could show that the FoP and depression persisted both in patients and caregivers over the study period. These results align with a study by Rooney, McNamara [17] on glioma patients, in which emotional distress persisted for at least 6 months after primary neurosurgery. Furthermore, depression symptoms have been linked to poorer attention and executive functioning in patients with primary brain tumors receiving radiotherapy, which could further impair patients' functional status over time [16]. Surprisingly, and contrary to our hypothesis, anxiety values decreased 6 months after diagnosis compared to the time of diagnosis. This reduction might be explained by a potential adaptation process experienced by both patients and caregivers, as they gradually adjust to the diagnosis. This aligns with concepts from models such as the Transactional Model and the Sense of Coherence Model [35, 36]. For example in young cancer patients, a reduction of anxiety over time was shown [37]. Descriptively however, this anxiety reduction from diagnosis to 6 months post‐diagnosis was only seen for caregivers, but not for the patient group. One possible explanation for this trend is that caregivers may experience significantly heightened anxiety at the time of diagnosis, reflecting an acute stress response to the sudden and overwhelming demands of caregiving. Over time, they may adapt to their roles, leading to a gradual reduction in anxiety. Targeted interventions especially for FoP and depression in this population might be worth investigating in future studies, to not only alleviate the psychological distress both in patients and their caregivers, but also to help prevent associated functional decline.

As our second hypothesis, we postulated that caregivers are at least as affected by the psychological distress as patients. For FoP, patients descriptively reported higher FoP at diagnosis and after 3 months, while caregivers reported higher FoP 6 months post‐diagnosis. Descriptively, the caregivers' anxiety and depression exceeded the patients' anxiety and depression at all time points. Our descriptive results are in line with the cross‐sectional study of Goebel, von Harscher [1], who identified psychiatric disorders in 47% of caregivers versus 38% of patients within the first three months of a primary brain tumor diagnosis. The multilevel analysis in our study revealed that caregivers experienced higher anxiety and depression at all time points and similar FoP compared with patients. These results are in contrast with a study of Braun, Aslanzadeh [13], who reported higher fear of cancer recurrence in caregivers compared with patients, however in a cross‐sectional design including solely primary brain tumors. With regards to the observed differences in the anxiety and depression between patients and caregivers, our study expands the existing literature by providing a longitudinal perspective on dyads in brain tumor cases, including those affected by secondary brain tumors. In previous qualitative studies, patients with primary brain tumors themselves have acknowledged the additional distress placed on their caregivers, highlighting the relevance of dyadic support [22, 38]. Compared with caregivers of patients with other cancer types [39], caregivers of patients with malignant brain tumors appear to be especially distressed. Our findings of heightened distress in caregivers could be attributed to the specific challenges associated with malignant brain tumors, such as a poor prognosis and personality changes [40]. Of note, quality of life in caregivers has been shown to be lower in cases of high‐grade disease [41]. Our results support and extend these previous findings, highlighting the additional distress of caregivers compared with patients. Future investigations are needed that specifically focus on caregivers' psychological needs and evaluate potential support strategies.

Finally, in our analysis, we found that taking into account the variability between the dyads was important for a sufficient description of the data. This aligns with the description of stress and coping in cancer patients and caregivers as interpersonal processes, framing cancer as a “we‐disease” [20]. Future research should investigate the longitudinal interrelationships between patient and caregiver distress in brain tumor dyads, enabling a more comprehensive understanding of their shared and individual psychological trajectories.

Regarding sociodemographic characteristics, our results are partially in line with previous studies. Like Goebel and Mehdorn [12], we found no effect of age or sex on FoP in patients. Regarding anxiety, previous studies reported female sex to be associated with higher anxiety [14] and lower mental health [18] in caregivers of brain tumor patients and with higher anxiety and depression in cancer patients overall [42]. Consistent with these findings, our study observed that female sex was associated with higher anxiety in our sample. However, no significant effect of sex was found for depression. Interestingly, older patients and caregivers reported higher depression than younger patients and caregivers. However, younger age has been linked to higher distress in neuro‐oncological caregivers [43] and glioma patients [17]. In our study, older participants could have been more advanced in their disease, potentially explaining the found differences between younger and older patients.

### 4.1 Study Limitations

The main limitation of our study is the high number of drop‐outs during the study period. However, as reported, no baseline differences between drop‐outs and included participants in outcomes were found. Therefore, minimal bias due to the drop‐outs can be assumed. Another limitation is the single center design and the assessment of a convenience clinical sample, limiting the generalizability of our results. Furthermore, participants designated caregivers for study participation in order to consider different social situations. Therefore, we included caregivers with different roles (e.g. partners, family members; Table 1) which could have potentially influenced results. Lastly, we have not considered all confounders reported in previous literature, such as social support and previous functional status of patients [18, 19]. However, we have included most of the components recommended by the Dyadic Cancer Outcomes Framework, such as patient and caregiver characteristics, the relationship characteristics and the cancer care trajectory [44]. Lastly, the participants included in our study differed significantly in terms of their prognostic situations (e.g., primary vs. secondary brain tumors, stable vs. progressive disease). To address this variability, we incorporated progression status as a factor in our analysis. However, it is important to note that the heterogeneity of the sample may limit the generalizability of our findings.

### 4.2 Clinical Implications

This study demonstrates the need for psychological support in patients with malignant brain tumors as well as in their caregivers. Furthermore, since the caregivers' reported higher anxiety and depression than patients and distress in patient‐caregiver dyads has been reported to be closely interrelated, interventions should focus on supporting caregivers to reduce future distress both in patients and caregivers [20]. In a study from Reblin, Small [19], the burden of caregivers at diagnosis was associated with lower social support at 4 months, linked to higher anxiety and depression after 8 months, potentially creating a negative cycle of burden and distress. So far however, evidence on psychological interventions in patients with brain tumors is limited and studies are often underpowered [45]. Additionally, interventions for caregivers are rarely supported by health insurances, as the German healthcare system primarily focuses on patient‐related cost reimbursement. Nevertheless, patients themselves agreed that caregiver support was at least equally important as patient support, and expressed that joint therapy sessions for patients and caregivers would be beneficial [22, 38]. A recent RCT of a home care intervention for patients with primary brain tumors and their caregivers observed promising results in reducing caregiver burden and improving patients' emotional and cognitive symptoms [46]. Importantly, anxiety and depression in patients with brain tumors have been associated with impaired neurocognition [16] and shorter survival rates [47], further emphasizing the clinical importance of interventions. Therefore, the psychological distress in patients with malignant brain tumors and their caregivers should be routinely assessed in clinical care. Future studies should explore psychological interventions aimed at reducing the psychological distress both in patients and caregivers.

## 5 Conclusion

This study was one of the first to assess the longitudinal course of FoP, anxiety and depression in dyads of patients with malignant brain tumors and their caregivers in the 6 months post‐diagnosis. We observed clinically relevant FoP, anxiety and depression in a significant percentage both of patients and caregivers. FoP and depression remained consistent over time, and anxiety decreased from diagnosis to 6 months post‐diagnosis. Additionally, caregivers were more affected by anxiety and depression at all time points. These results support the evaluation and treatment of FoP, anxiety and depression in patients with malignant brain tumors and their caregivers. We recommend future clinical trials to investigate psychological interventions that support patients with malignant brain tumors and their caregivers conjointly.

## Author Contributions

Conceptualization: A.K., A.‐M.K., M.R. Data curation: A.‐M.K. Formal analysis: A.K., A.‐M.K., R.S., M.R. Funding acquisition: A.K., M.R. Investigation: A.‐M. K., C.Q., M.R. Methodology: A.‐M.K., R.S., M.R., A.K. Project administration: A.K., A.‐M.K., C. Q., M.R. Resources: A.K., M.S., M.R. Supervision: A.K., M.R. Validation: A.K., M.R. Visualization: A.‐M. K., M.K.K. Writing–original draft: A.‐M.K., M.K.K., A.K. Writing–review & editing: A.K., A.‐M.K, C.Q., M.K.K., R.S., M.S., M.R. All authors approved the submitted version of the article and agreed to be accountable for all aspects of the work.

## Ethics Statement

This study was approved by the ethics committee of the Medical Faculty at Heinrich‐Heine‐University Duesseldorf (Ethic ID: 2018‐338‐ProspDEuA). The study was conducted in accordance with legal and regulatory requirements, as well as the general principles set forth in the Declaration of Helsinki, §15 of the German Medical Association's professional code of conduct “Berufsordnung für Ärzte, BOÄ”, and the applicable data protection law. All participants were informed about the study design and gave written informed consent before being included in the study.

## Conflicts of Interest

The authors declare no conflicts of interest.

## Data Availability

The data that support the findings of this study are available on request from the corresponding author. The data are not publicly available due to privacy or ethical restrictions.
